# Determinants of smoking prevention behavior of senior high school students: A short report

**DOI:** 10.18332/tid/200748

**Published:** 2025-03-19

**Authors:** Muthmainnah Muthmainnah, Galuh Mega Kurnia, Avinka Nugrahani

**Affiliations:** 1 Department of Epidemiology, Biostatistics and Demography, Health Promotion and Behavioral Science, Faculty of Public Health, Universitas Airlangga, Surabaya, Indonesia; 2 Faculty of Public Health, Universitas Airlangga, Surabaya, Indonesia; 3 Department of Environmental Health, Faculty of Public Health, Universitas Airlangga, Surabaya, Indonesia

**Keywords:** public health, health belief model, school, smoking prevention, adolescent

## Abstract

**INTRODUCTION:**

With Indonesia ranking top in the Association of Southeast Asian Nations for the number of smokers aged 13–15 years, this study aims to analyze the factors associated with smoking prevention behavior among students of senior high school.

**METHODS:**

This cross-sectional pilot study, conducted in 2022 with 90 samples of grade 10–11 students at SMA Negeri 1 Taman Sidoarjo East Java Indonesia, examined variables such as perceived vulnerability (the belief about the risk of experiencing a health issue), severity (the belief about the seriousness of the health issue), benefits (the belief in the benefit of taking preventive actions), barriers (the perceived obstacles to performing preventive behaviors), self-efficacy (the confidence in one's ability to perform preventive behaviors successfully), and cues to action (factors that trigger the decision to engage) in relation to health behaviors. Data were analyzed using the chi-squared test.

**RESULTS:**

The chi-squared analysis showed significant associations between several factors and smoking prevention behavior. For perceived susceptibility, 34.4% with high susceptibility had good behavior, and 13.3% had not good behavior (p=0.000). For perceived severity, 33.3% with high severity exhibited good behavior, and 21% had not good behavior (p=0.002). Regarding perceived benefits, 28.9% with high benefits showed good behavior, while 22.6% had not good behavior (p=0.018). Self-efficacy indicated 36.7% with high self-efficacy demonstrated good behavior versus 25.8% with not good behavior (p=0.001). Cues to action revealed that 28.9% with high cues had good behavior, and 18.9% did not have good behavior (p=0.003). No association was found for perceived barriers (p=0.386).

**CONCLUSIONS:**

The level of smoking prevention behavior is influenced by perceived susceptibility, severity, benefits, self-efficacy, and cues to action. Therefore, more intensive and targeted efforts are needed to promote awareness of the dangers of smoking and to enhance adolescents' self-efficacy in preventing smoking.

## INTRODUCTION

In Indonesia, smoking among adolescents is a significant concern, with the Southeast Asia Tobacco Control Alliance reporting high smoking rates, particularly among males aged 13–15 years^1^. The 2019 Global Youth Tobacco Survey showed 19.2% of students currently smoke^2^, with a significant increase in smoking prevalence among those aged 15–19 years, from 2013 to 2018 by 1.4%^3^. In East Java, 9.84% of adolescents aged 10–18 years smoke, and in Sidoarjo District, 20.46% of the population aged ≥10 years smoke daily^4^.

Peer influence is a significant risk factor for adolescent smoking, with studies showing that peer smoking behavior can increase the likelihood of smoking by up to 10-fold^5,6^. Smoking at a young age increases the risk of long-term health problems due to prolonged exposure to tobacco toxins^6^. Health Belief Model (HBM) theory, which focuses on factors such as perceived susceptibility, severity, self-efficacy, benefits, and barriers, is commonly used to analyze health behavior change^7^. Previous research has shown that HBM-based interventions, such as smoking prevention counseling, can positively influence adolescents’ perceptions of smoking risks and benefits. However, some adolescents with low perceptions of smoking’s risks may overlook its harms^8^. This study aims to analyze factors associated with smoking prevention behavior among students at a senior high school in Indonesia to help design more effective smoking prevention interventions.

## METHODS

This study in 2022 was located in Sidoarjo Regency, Indonesia, and used a cross-sectional method. The population in this study consisted of students from SMA Negeri 1 Taman Sidoarjo East Java Indonesia, specifically those in grades 10 and 11. The total population of grade 10 and 11 students was 727, with 382 students in grade 10 and 345 in grade 11. This population was chosen because smoking behavior is most prevalent in the high school age group. Grade 12 students were excluded from the study as they were focused on preparing for national exams. Population data were obtained from secondary data related to the number of students in the school. The sampling method used in this study was convenience sampling, where questionnaire links were distributed to class leaders and then shared with their classmates. The final sample of 90 students was drawn from this population of Senior High School 1 Taman students.

### Measures

The questionnaire was developed based on the Health Belief Model (HBM) theory and consists of seven main domains: perceived susceptibility, perceived severity, self-efficacy, perceived benefits, perceived barriers, cues to action, and prevention behavior. The questionnaire was tested for validity and reliability on 30 respondents at a different school with similar characteristics. Each domain was assessed using specific items on an ordinal scale, and the results were categorized based on the mean value (high >mean, and low ≤mean). The following is a detailed explanation of each domain:

1. Perceived susceptibility: This domain measures respondents’ perceptions of their risk of developing health problems due to smoking behavior. It consists of five questionnaire items assessed on an ordinal scale.
2. Perceived severity: This domain evaluates respondents’ perceptions of the seriousness of health threats caused by smoking. It includes five questionnaire items on an ordinal scale.
3. Self-efficacy: This domain assesses respondents’ confidence in preventing smoking or resisting smoking-related temptations. It comprises six questionnaire items measured on an ordinal scale.
4. Perceived benefits: This domain captures respondents’ perceptions of the effectiveness of smoking prevention behaviors. It includes five questionnaire items on an ordinal scale.
5. Perceived barriers: This domain identifies respondents’ obstacles to adopting smoking prevention behaviors. It consists of five questionnaire items measured on an ordinal scale.
6. Cues to action: This domain examines respondents’ responses to cues or stimuli that encourage smoking prevention behaviors. It includes five questionnaire items on an ordinal scale.
7. Prevention behavior: This domain evaluates the efforts made by respondents to avoid smoking and its associated risks. It consists of six questionnaire items assessed on an ordinal scale.

The time required to complete the questionnaire was approximately 10–15 minutes, and participants’ identities were kept strictly confidential to ensure privacy and encourage honest responses. The questionnaire is provided in the Supplementary file.

### Statistical analysis

This study uses the chi-squared test to analyze the association between the independent and dependent variables. Data analysis was carried out with IBM SPSS Statistics (Version 18).

## RESULTS

**Figure 1:**
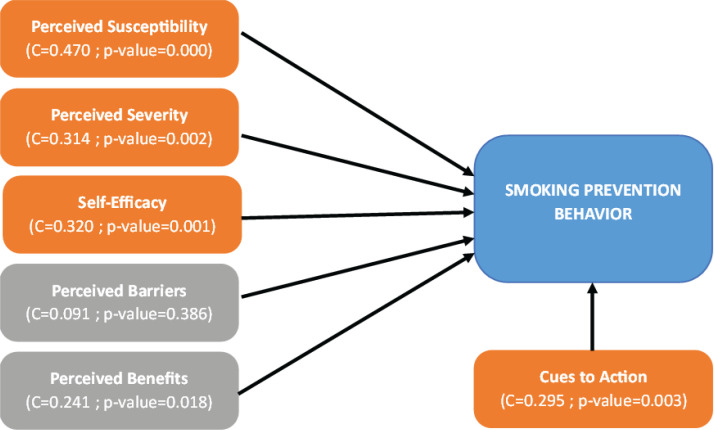
Factors influencing smoking prevention behavior

The cross-tabulation results show that the perceived vulnerability variable has a positive correlation with smoking prevention behavior (C=0.470; p=0.000) (Table 1 and Figure 1). Similarly, on the perceived severity variable, a positive association was found with smoking prevention behavior (C=0.314; p=0.002). The results also showed that on the perceived benefits variable, there was a positive association with smoking prevention behavior (C=0.241; p=0.018). However, for the perceived barriers variable, no correlation was found with smoking prevention behavior (C=0.091; p=0.386). In addition, in the self-efficacy variable, an association was found with smoking prevention behavior (C=0.320; p=0.001). Finally, the cues to action variable also showed a positive association with smoking prevention behavior (C=0.295; p=0.003).

**Table 1:** Chi-squared analysis of factors associated with smoking prevention behavior

*Variable*		*Smoking prevention behavior*				*Total*		*Mean*	p*	*Contingency coefficient (C)*
	*Good*		*Not good*							
	*n*	*%*	*n*	*%*	*n*	*%*				
Perceived susceptibility	High	31	34.4	12	13.3	43	47.8	17.122	0.000	0.470
	Low	9	10	38	42.2	47	52.5			
Perceived severity	High	30	33.3	21	23.3	51	56.7	17.31	0.002	0.314
	Low	10	11.1	29	32.2	39	43.3			
Perceived benefit	High	26	28.9	20	22.2	46	51.1	21	0.018	0.241
	Low	14	15.6	30	33.3	44	48.9			
Perceived barriers	High	26	28.9	28	31.1	54	60	15	0.386	0.091
	Low	14	15.6	22	24.4	36	40			
Self-efficacy	High	33	36.7	25	27.8	58	64.4	26.22	0.001	0.320
	Low	7	7.8	25	27.8	32	35.6			
Cues to action	High	26	28.9	17	18.9	43	47.8	18	0.003	0.295
	Low	14	15.6	33	36.7	47	52.2			

High/good: >mean; low/not good: ≤mean; mean=4.

* p-value: Fisher’s exact test, significant if <0.005.

## DISCUSSION

The perceived vulnerability was positively correlated with smoking prevention behavior. Students who perceive a higher risk of smoking-related diseases tend to adopt better prevention behaviors. This suggests that greater awareness of health risks enhances the likelihood of taking preventive actions. Previous studies also show that adolescents who view smoking as a stress reliever are more likely to smoke, indicating that perceptions of smoking’s benefits can influence vulnerability to smoking^9^. Perceived severity was also positively associated with smoking prevention behavior. Students who perceive smoking-related diseases as serious are more motivated to engage in preventive actions. The recognition of the significant negative impacts of smoking can drive individuals to prioritize prevention over treatment^10^.

Similarly, perceived benefits correlated positively with smoking prevention behavior. Students who see the benefits of smoking prevention are more likely to adopt preventive behaviors. When choosing health behaviors, people often weigh the benefits against the availability of resources and environmental support^11^. On the other hand, perceived barriers did not show a positive correlation with smoking prevention behavior, contradicting previous studies suggesting that higher perceived barriers reduce smoking intentions. This finding indicates the importance of strategies that reduce the perceived barriers to prevention, such as increasing cigarette prices and taxes to limit access^12,13^.

Self-efficacy was positively correlated with smoking prevention behavior. Students with high self-efficacy are more likely to engage in prevention behaviors, and their confidence in their ability to succeed in avoiding smoking is an essential factor. Programs targeting smoking prevention can benefit from fostering self-efficacy, such as by establishing anti-smoking student cadres in schools^14^. Cues to action, both internal and external, were also positively related to smoking prevention behavior. Students who are exposed to cues, like peer influence or media messages, are more likely to adopt smoking prevention behaviors. The presence of smoking peers and parental smoking behaviors can serve as triggers for initiating or maintaining smoking habits^15,16^.

### Strengths and limitations

This study addresses a significant public health issue in Indonesia, namely the high prevalence of adolescent smoking. This study explores the factors that influence smoking prevention behavior among high school students and provides valuable insights for smoking prevention efforts at the adolescent level. The findings provide a strong basis for the development of smoking prevention policies in Indonesian high schools. Identifying factors that influence smoking prevention behavior can help design more effective interventions.

This study has several limitations. First, there is a potential for respondent bias due to reliance on self-reported data from students, including recall bias or the tendency to provide socially desirable answers, which may reduce the validity of the results. Second, the study was conducted in only one high school in Indonesia, limiting its generalizability to other regions or countries. Third, the study lacks adjusted comparisons using regression models, which could provide a more robust analysis. Additionally, residual confounding may still exist, and there are empty categories in certain variables, such as female former smokers, which limit the comprehensiveness of the analysis. Despite extensive research on the HBM in smoking prevention, no prior study has specifically analyzed adolescent smoking behavior using this model at this school. Future studies should address these limitations to enhance the reliability and applicability of findings.

## CONCLUSIONS

The majority of smoking prevention behaviors among students of Senior High School 1 Taman were rated as suboptimal. Relationships were found between perceived susceptibility, severity, benefits, self-efficacy, and cues to action with adolescent smoking prevention behavior. However, no correlation was found between perceived barriers and smoking prevention behavior among adolescents at Senior High School 1 Taman in Indonesia.

## Supplementary Material



## Data Availability

Data supporting this research are available, through a request with a clear reason, from the authors. To protect respondents, research data will be destroyed two years after the research is completed.
