# Supplementary material for: Determinants of smoking prevention behavior of senior high school students: A short report
Source: Tob Induc Dis. 2025 Mar 19;23:10.18332/tid/200748. doi: 10.18332/tid/200748 (PMC11921431; doi:10.18332/tid/200748)
Supplement: Supplementary file 1 [file TID-23-37-s1.pdf]

Supplementary file.

Frequency Distribution Based on Modifying Factors

Table 1. Frequency Distribution of Respondent Characteristics

Characteristics	Frequency (n = 90)	Percentage (%)
**Age**		
15 – 17 years old	81	90
18 – 21 years old	9	10
**Sex**		
Male	32	35.6
Female	58	64.4
**Class**		
X	47	52.2
XI	43	47.8
**Father's Education Level**		
Elementary school/equivalent	5	5.6
Junior high school/equivalent	13	14.4
Senior high school/equivalent	50	55.6
**Daily Allowance**		
Below IDR 10,000	45	50
IDR 10,000 to IDR 20,000	38	42.2
More than IDR 20,000	7	7.8

Table 1. shows that the age of respondents is divided into two, namely middle adolescence as many as 81 students and late adolescence as many as 9 students. There were 32 male students and 58 female students. A total of 47 students came from grade 10 and 43 students came from grade 11. The pocket money that students have per day is below Rp10,000, namely 45 students.

Table 2: Frequency Distribution of Smoking Prevention Behavior

Smoking Prevention Behavior	Frequency (n = 90)	Percentage (%)
Good	40	44.4
Not Good	50	55.6

Good: >Mean; Not Good: ≤ Mean

Table 2. shows that most students have a level of smoking prevention behavior in the less category, namely 50 students (55.6%). There were 40 students (44.4%) who had good smoking prevention behavior.

QUESTIONNAIRE

RESPONDENT IDENTITY

1. Respondent Name :
2. Age :
3. Gender :
   a. Male
   b. Female
4. Class :
   a. X
   b. XI

MODIFYING FACTORS

1. Father's Education Level :
   a. Not in School
   b. Elementary School / Equivalent
   c. Junior High School / Equivalent
   d. Senior High School / Equivalent
   e. Diploma
   f. Bachelor
2. Mother's Education Level :
   a. Not in School
   b. Elementary School / Equivalent
   c. Junior High School / Equivalent
   d. Senior High School / Equivalent
   e. Diploma
   f. Bachelor
3. Father's occupation :
   a. Not Working
   b. Labor
   c. Entrepreneur
   d. Teacher or Lecturer

e. Civil Servants

f. Private employee

g. More

4. Mother's Occupation :

   a. Not Working

   b. Labor

   c. Entrepreneur

   d. Teacher or Lecturer

   e. Civil Servants

   f. Private employee

   g. More

5. Father's Income :
6. Mother's Income :
7. Allowance per Day :
8. Knowledge

No.	Statement	Answer	
		Correct	Wrong
1.	Habit smoking can result in chronic lung disease, tooth decay, cancer skin, and heart attack		
2.	Cigarettes can cause addiction or addiction		
3.	People who do not smoke but inhale cigarette smoke when around people who smoke, will not be affected in any way to his health		
4.	Cigarette smoke exposure does not pose a risk for pregnant women		
5.	Tar is the only toxic material that contained in cigarettes		
6.	Adverse health effects of active smokers smaller than secondhand smoke		
7.	SFA (Smoke Free Area) is a regulation that allows smoking in public places		
8.	Nicotine is the most harmful substance in cigarettes		
9.	Tooth decay is a long-term effect short of smoking		
10.	Cigarette smoke inhalers are 14 times more likely to suffer from lung cancer than people who do not smoke cigarettes		

PERCEIVED VULNERABILITY

SA: Strongly Agree

A: Agree

D: Disagree

SD: Strongly Disagree

No.	Question	Answer			
		SA	A	D	SD
1.	According to me, habit smoking can increases the risk of developing lung cancer				
2.	If I smoke, it will have an impact negative for my health and that of the people who breathe in the smoke				
3.	If a family member smokes in the house, children are at risk in respiratory disease				
4.	If I don't smoke, it will reduce my risk of lung cancer, stroke, high blood pressure, and other diseases,				
5.	In my opinion, smoking does cause any impact on health				

PERCEIVED SEVERITY

SA: Strongly Agree

A: Agree

D: Disagree

SD: Strongly Disagree

No.	Question	Answer			
		SA	A	D	SD
1.	Cigarette smoke inhaled by other people (passive smokers) will have a more dangerous impact for the health of the person compared to active smokers				
2.	I'm concerned that smoking can lead to the risk of developing lung cancer, heart attacks, and even passed away				
3.	In my opinion, by smoking a maximum of 1 sticks per day will not cause adverse health effects				

No.	Question	Answer			
		SA	A	D	SD
4.	In my opinion, lung cancer that occurs due to smoking can be treated with easy				
5.	In my opinion, diseases caused by smoking will hinder daily activities				

PERCEIVED BENEFITS

SA: Strongly Agree

A: Agree

D: Disagree

SD: Strongly Disagree

No.	Question	Answer			
		SA	A	D	SD
1.	In my opinion, smoking can eliminate stress and makes me calm down				
2.	In my opinion, by doing positive activities such as reading books and strengthening worship will be beneficial to avoid smoking behavior				
3.	In my opinion, with a warning picture the dangers of smoking in advertisements will be beneficial for strengthening smoking prevention				
4.	In my opinion, the provision of education from schools, families, and health workers will be useful for smoking prevention efforts.				
5.	I think smoking can help me confident to interact with others				
6.	In my opinion, smoking makes me more being accepted in a friendship environment				

PERCEIVED BARRIERS

SA: Strongly Agree

A: Agree

D: Disagree

SD: Strongly Disagree

No.	Question	Answer			
		SA	A	D	SD
1.	Hanging out with friends who smoke an obstacle to not smoking				
2.	I think a family environment that smokes an obstacle to not smoking				
3.	The stressful or depressed state I feel will be an obstacle me to not smoking				
4.	If someone offered me cigarettes, it would be a deterrent for me to not smoke.				
5.	If I don't smoke then I won't accepted in my circle of friends				

SELF-EFFICACY

VS : Very Sure

S : Sure

NS: Not Sure

VU: Very Unsure

No.	Question	Answer			
		VS	S	NS	VU
1.	I believe that I can cope with stress that I feel even without smoking				
2.	I feel sure and confident my ability to deal with problems that occur even without smoking				
3.	I believe that by not smoking I can have a positive impact on me and the people around me				
4.	I believe that even though I don't smoke I wil keep accepted by my friendship environment				
5.	I believe that I can be a role model to my family or neighborhood if I don't smoke				
6.	I believe that choosing not to smoke is an informed decision or choice right				
7.	I am confident that I will not smoke despite being in an environment that encouraged me to smoke				

CUES TO ACTION

SA: Strongly Agree

A: Agree

D: Disagree

SD: Strongly Disagree

No.	Question	Answer			
		SA	A	D	SD
1.	My family gave me advice to stay away from smoking behavior				
2.	My friend supports me to stay away smoking behavior				
3.	Health Officer provide information on the dangers of smoking and efforts to smoking prevention				
4.	The teacher provides information about the dangers of smoking and smoking prevention efforts				
5.	I don't care about anything regarding the dangers of smoking and ways prevention smoking				

SMOKING PREVENTION BEHAVIOR

No.	Question	Answer	
		Yes	No
1.	I have searched for information about the dangers of smoking		
2.	I've been looking for information on how to avoid smoking and how to quit smoking		
3.	I received education on smoking prevention from school		
4.	I dare to reprimand people who smoke in public places or in SFA (Smoke Free Area)		
5.	I dare to reprimand people who try to influencing others to smoke		
6.	I am willing to provide information regarding the dangers of smoking and smoking prevention measures to others		

KUESIONER

IDENTITAS RESPONDEN

1. Nama Responden :
2. Usia :
3. Jenis Kelamin :
   a. Laki-laki
   b. Perempuan
4. Kelas :
   a. X
   b. XI

MODIFYING FACTORS

1. Tingkat Pendidikan Ayah :
   a. Tidak Sekolah
   b. SD
   c. SMP
   d. SMA/Sederajat
   e. D3
   f. Sarjana
2. Tingkat Pendidikan Ibu :
   a. Tidak Sekolah
   b. SD
   c. SMP
   d. SMA/Sederajat
   e. D3
   f. Sarjana
3. Pekerjaan Ayah :
   a. Tidak Bekerja
   b. Buruh
   c. Pengusaha
   d. Guru atau Dosen
   e. Pegawai Negeri Sipil (PNS)

f. Karyawan swasta

g. Lainnya

4. Pekerjaan Ibu :

a. Tidak Bekerja

b. Buruh

c. Pengusaha

d. Guru atau Dosen

e. Pegawai Negeri Sipil (PNS)

f. Karyawan swasta

g. Lainnya

5. Pendapatan Ayah :

6. Pendapatan Ibu :

7. Uang saku per Hari :

8. Pengetahuan

No	Pernyataan	Jawaban	
		**Benar**	**Salah**
1.	Kebiasaan merokok dapat mengakibatkan penyakit paru-paru kronis, merusak gigi, kanker kulit, dan serangan jantung		
2.	Rokok dapat menyebabkan ketagihan atau kecanduan		
3.	Orang yang tidak merokok namun menghirup asap rokok ketika berada di sekitar orang yang merokok, tidak akan terkena dampak apapun terhadap kesehatannya		
4.	Paparan asap rokok tidak menimbulkan risiko bagi ibu hamil		
5.	Tar hanya satu-satunya bahan beracun yang terdapat di dalam rokok		
6.	Dampak buruk bagi kesehatan perokok aktif lebih kecil daripada perokok pasif		
7.	KTR (Kawasan Tanpa Rokok) merupakan peraturan boleh merokok di tempat umum		
8.	Nikotin adalah zat yang paling berbahaya pada rokok		
9.	Kerusakan pada gigi merupakan efek jangka pendek dari merokok		
10.	Penghisap asap rokok berisiko 14 kali menderita kanker paru-paru dibandingkan orang yang tidak menghisap asap rokok		

KERENTANAN YANG DIRASAKAN

SS : Sangat Setuju S :

Setuju

TS : Tidak Setuju

STS : Sangat Tidak Setuju

No	Pertanyaan	Jawaban			
		STS	TS	S	SS
1.	Menurut saya, kebiasaan merokok dapat meningkatkan risiko terkena kanker paru-paru				
2.	Jika saya merokok, akan menimbulkan dampak negatif bagi kesehatan saya dan orang yang menghirup asap rokok tersebut				
3.	Jika ada anggota keluarga yang merokok di dalam rumah maka akan berisiko anak-anak mengalami penyakit ISPA				
4.	Jika saya tidak merokok maka akan mengurangi risiko terkena dari kanker paru, stroke, dan tekanan darah tinggi				
5.	Menurut saya, merokok tidak menimbulkan dampak apapun bagi kesehatan				

KEPARAHAN YANG DIRASAKAN

SS : Sangat Setuju S :

Setuju

TS : Tidak Setuju

STS : Sangat Tidak Setuju

No	Pertanyaan	Jawaban			
		STS	TS	S	SS
1.	Asap rokok yang dihirup oleh orang lain (perokok pasif) akan menimbulkan dampak yang lebih berbahaya bagi kesehatan orang tersebut dibandingkan perokok aktif				
2.	Saya khawatir bahwa merokok dapat menyebabkan risiko terkena penyakit kanker paru-paru, serangan jantung, dan bahkan meninggal dunia				
3.	Menurut saya, dengan merokok maksimal 1				

No	Pertanyaan	Jawaban			
		STS	TS	S	SS
	batang per hari tidak akan menimbulkan dampak buruk bagi kesehatan				
4.	Menurut saya, penyakit kanker paru-paru yang terjadi akibat merokok dapat diobati dengan mudah				
5.	Menurut saya, penyakit yang ditimbulkan dari merokok akan menghambat aktivitas sehari-hari				

MANFAAT YANG DIRASAKAN

SS : Sangat Setuju S :

Setuju

TS : Tidak Setuju

STS : Sangat Tidak Setuju

No	Pertanyaan	Jawaban			
		STS	TS	S	SS
1.	Menurut saya, merokok dapat menghilangkan stres dan membuat saya menjadi tenang				
2.	Menurut saya, dengan melakukan kegiatan positif seperti membaca buku dan memperkuat ibadah akan bermanfaat untuk menghindari perilaku merokok				
3.	Menurut saya, dengan adanya gambar peringatan bahaya merokok di iklan akan bermanfaat untuk memperkuat pencegahan merokok				
4.	Menurut saya, adanya pemberian edukasi dari pihak sekolah, keluarga, dan petugas kesehatan akan bermanfaat untuk upaya pencegahan merokok				
5.	Menurut saya, merokok dapat membantu saya percaya diri untuk berinteraksi dengan orang lain				
6.	Menurut saya, merokok membuat saya akan lebih diterima di lingkungan pertemanan				

HAMBATAN YANG DIRASAKAN

SS : Sangat Setuju S :

Setuju

TS : Tidak Setuju

STS : Sangat Tidak Setuju

No	Pertanyaan	Jawaban			
		STS	TS	S	SS
1.	Berkumpul dengan teman-teman yang merokok merupakan hambatan untuk tidak merokok				
2.	Menurut saya lingkungan keluarga yang merokok merupakan hambatan untuk tidak merokok				
3.	Keadaan stres atau tertekan yang saya rasakan akan menjadi penghambat saya untuk tidak merokok				
4.	Jika ada seseorang yang menawarkan rokok kepada saya maka akan menjadi penghambat saya untuk tidak merokok				
5.	Jika saya tidak merokok maka saya tidak akan diterima di lingkungan pertemanan saya				

EFIKASI DIRI

SY : Sangat Yakin Y :

Yakin

TY : Tidak Yakin

STY : Sangat Tidak Yakin

No	Pertanyaan	Jawaban			
		STY	TY	Y	SY
1.	Saya yakin bahwa saya dapat mengatasi stres yang saya rasakan walaupun tanpa rokok				
2.	Saya merasa yakin dan percaya diri terhadap kemampuan yang saya miliki dalam menghadapi permasalahan yang terjadi walaupun tanpa rokok				
3.	Saya yakin dengan tidak merokok saya dapat				

No	Pertanyaan	Jawaban			
		STY	TY	Y	SY
	memberikan dampak yang positif bagi saya dan orang di sekitar saya				
4.	Saya yakin bahwa meskipun saya tidak merokok saya akan tetap diterima oleh lingkungan pertemanan saya				
5.	Saya yakin bahwa saya dapat menjadi panutan bagi keluarga atau lingkungan sekitar saya jika saya tidak merokok				
6.	Saya yakin bahwa memilih untuk tidak merokok merupakan suatu keputusan atau pilihan yang tepat				
7.	Saya yakin bahwa saya tidak akan merokok walaupun berada di lingkungan yang mendorong saya untuk merokok				

ISYARAT UNTUK BERTINDAK

SS : Sangat Setuju S :

Setuju

TS : Tidak Setuju

STS : Sangat Tidak Setuju

No	Pertanyaan	Jawaban			
		STS	TS	S	SS
1.	Keluarga memberikan nasihat kepada saya untuk menjauhi perilaku merokok				
2.	Teman saya mendukung saya untuk menjauh perilaku merokok				
3.	Petugas kesehatan memberikan informasi mengenai bahaya merokok dan upaya untuk pencegahan merokok				
4.	Guru memberikan informasi mengenai bahaya merokok dan upaya pencegahan merokok				
5.	Saya tidak peduli dengan apapun mengenai bahaya merokok dan cara-cara pencegahan merokok				

PERILAKU PENCEGAHAN MEROKOK

No	Pertanyaan	Jawaban	
		Ya	Tidak
1.	Saya pernah mencari informasi mengenai bahaya merokok		
2.	Saya pernah mencari informasi mengenai bagaimana cara yang dapat dilakukan untuk menghindari kebiasaan merokok dan cara untuk berhenti merokok		
3.	Saya mendapatkan edukasi pencegahan merokok dari Sekolah		
4.	Saya berani menegur orang yang merokok di tempat umum atau di KTR		
5.	Saya berani menegur orang yang berusaha untuk mempengaruhi orang lain untuk merokok		
6.	Saya bersedia memberikan informasi mengenai bahaya merokok dan upaya pencegahan merokok kepada orang lain		

© 2025 Muthmainnah M. et al.
